# A mathematic model to reveal delicate cross‐regulation between MAVS/STING, inflammasome and MyD88‐dependent type I interferon signalling

**DOI:** 10.1111/jcmm.15768

**Published:** 2020-09-03

**Authors:** Chunmei Cai, Xiao Yu

**Affiliations:** ^1^ Research Center for High Altitude Medicine School of Medical Qinghai University Xining China; ^2^ Key Laboratory of Application and Foundation for High Altitude Medicine Research in Qinghai Province Xining China; ^3^ Department of Immunology School of Basic Medical Sciences Southern Medical University Guangzhou China; ^4^ Guangdong Provincial Key Lab of Single Cell Technology and Application Southern Medical University Guangzhou China

**Keywords:** delicate cross‐regulation, malaria, mathematical model, type I interferon

## Abstract

Early type I interferon is essential for antagonizing against malaria infection, which remains a significant global infectious disease. After *Plasmodium yoelii* YM infection, the activation of MAVS‐, STING‐ and inflammasome‐IRF3‐mediated pathway could trigger the *Socs1* expression to inhibit the TLR7‐MyD88‐IRF7‐induced type I interferon production. However, the dynamic regulatory mechanisms of type I interferon response to YM infection and delicate cross‐regulation of these signalling are far from clear. In current study, we established a mathematical model to systematically demonstrate that the MAVS‐, STING‐ and inflammasome‐mediated signalling pathways play distinct roles in regulating type I interferon response after YM infection; and the YM dose could significantly affect the difference of resistance to YM infection among MAVS, STING and inflammasome deficiency. Collectively, our study systematically elucidated the precise regulatory mechanisms of type I interferon signalling after YM infection and advanced the research on therapy of plasmodium infection by incorporating multiple signalling pathways at diverse time.

## 1 INTRODUCTION

The innate immune response, triggered by pathogen‐associated molecular patterns (PAMPs), serves as the first line to provide powerful defence against invading microbes.^1^, ^2^, ^3^ The specific recognition of PAMPs depends on kinds of pattern recognition receptors (PRRs), including Toll‐like receptors (TLRs), NOD‐like receptors (NLRs), RIG‐I‐like receptors (RLRs), C‐type lectin receptors and DNA sensors.^4^, ^5^, ^6^, ^7^, ^8^, ^9^ Activation of these receptors by binding with diverse ligands generally converges on common downstream pathways, such as type I interferon, NF‐κB and inflammasome signalling.^9^, ^10^, ^11^, ^12^, ^13^, ^14^ Hence, the stringent regulation of these common pathways is required for orchestrating effective innate immune response. However, the relative contribution of these pathways in response to malaria infection remains be further defined.

Malaria is a deadly infectious disease that affects approximately 200 million people (WHO2019), leading to about half a million deaths each year.^15^, ^16^, ^17^ No highly effective vaccine has been a major limiting factor in preventing malaria infection, which largely arise from the incomplete understanding of the underlying mechanism of host‐parasite interactions.^18^, ^19^, ^20^, ^21^, ^22^ Malaria infection initiates a systemic immune response and subsequently triggers a elevated release of inflammatory cytokines, resulting in parasite elimination and/or disease.^22^, ^23^, ^24^, ^25^, ^26^, ^27^, ^28^, ^29^ Our previous studies showed that during *P.lasmodium yoelii* YM (YM for short) strain blood stage infection, several components of malaria, including haemozoin, genomic DNA (gDNA) and RNA, could simultaneously activate diverse host sensors to initiate multiple pathways activation.^30^, ^31^ Although it is conceivable that the MAVS‐, STING‐, and inflammasome‐mediated signalling pathways converge to induce SOCS1 expression to modulate TLR7‐MyD88‐IRF7‐dependent type I IFN response during malaria infection, there is still lack of systematic analysis of underlying mechanisms in (a) what exact role does the MAVS‐, STING‐ or inflammasome‐mediated pathway play in anti‐malaria immunity, and (b) how these dynamic networks cooperatively response to varying dose or time of YM challenging.

In this study, we developed a mathematical model to quantitatively and systematically investigate the underlying mechanisms involved in the dynamic regulation of MAVS/STING, inflammasome and MyD88‐dependent type I interferon responses to YM challenging. We demonstrated that the properties of MAVS‐, STING‐, and Inflammasome‐mediated signalling pathways have diverse impact on *Socs1* and *Ifnα/β* expression by YM treatment. Besides, we identified that mice deficient in MAVS‐, STING‐ or inflammasome‐mediated pathways also have distinct resistance to YM infection. Interestingly, we found that the YM dose could significantly affect the difference of resistance to YM infection among MAVS, STING and Caspase1 deficiency. Our findings further revealed that the synergistic or antagonistic effect of these three pathways on *Socs1* or *Ifnα/β* expression is also diverse for varying time and stimulus, respectively.

## 2 MATERIAL AND METHODS

### 2.1 Microbes

The *Plasmodium yoelii* YM has been previously described.^32^

### 2.2 Primary cells

Bone marrow cells were isolated from the tibia and femur, and cultured in RPMI1640 medium with 10% FBS, 1% penicillin‐streptomycin and 200 ng/mL Flt3L for 7 days to harvest pDCs.

### 2.3 Animals

Female mice of C57BL/6 (WT), *Aim2^‐/‐^*, *Nlrp3^‐/‐^*, *Casp1^‐/‐^*, *Il1r1^‐/‐^*, *Tmem173^gt^* and *Mavs^‐/‐^* were purchased from the Jackson Laboratory. All animal studies were approved by the ethics committee of Qinghai University and carried out in accordance with animal management regulations of the Ministry of Health of China.

### 2.4 Isolation and preparation of *Plasmodium* gDNA, RNA and haemozoin

Parasite‐infected mice blood was collected in saline solution and filtered to deplete white blood cells. Parasites were spun down after RBC lysis buffer treatment, and lysate incubated with buffer A (150 mmol/L NaCl, 25 mmol/L EDTA, 10% SDS and protein kinase) overnight. gDNAs were isolated using phenol/chloroform, and RNAs were isolated using TRIzol reagent (Invitrogen). Haemozoin was purified as previously described.^28^

### 2.5 RNA extraction and quantitative polymerase chain reaction (qPCR)

Total RNA was isolated from primary cells with TRIzol reagent (Invitrogen), according to the manufacturer's instructions. The complimentary cDNA was performed using reverse transcriptase IV (Invitrogen). Quantitative real‐time PCR was performed using the ABI Prism 7000 analyser (Applied Biosystems), and using iTaq SYBR Green Supermix (BioRad) with following specific primers:

*mIfnα* forward: GGACTTTGGATTCCCGCAGGAGAAG.

*mIfnα* reverse: GCTGCATCAGACAGCCTTGCAGGTC.

*mIfnβ* forward: TCACCTACAGGGCGGACTTC.

*mIfnβ* reverse: TCTCTGCTCGGACCACCATC.

*mSocs1* forward: CTGCGGCTTCTATTGGGGAC.

*mSocs1* reverse: AAAAGGCAGTCGAAGGTCTCG.

### 2.6 Mathematical modelling

We developed a simplified model to describe STING‐, MAVS‐ and inflammasome‐mediated *Socs1* expression, which negatively regulates type I IFN response to YM challenge in pDCs. Several components of malaria could simultaneously activate diverse host sensors to initiate immune response.^24^, ^30^ Activation of MDA5 and an unrevealed RNA sensor recruits the MAVS adaptor protein, whereas stimulation of DNA sensors (cGAS and an unrevealed DNA sensor) leads to the recruitment of STING, which both induce the phosphorylation of IRF3 (reactions (2), (3), (4), (5), 11‐12 in Table S1).^30^, ^33^, ^34^ In addition, we have found that the gDNA‐haemozoin complex could activate the AIM2 and NLRP3 inflammasomes, respectively, to initiate the IL‐1β signalling to activate IRF3 in pDCs (reactions (6), (7), (8), (9), (10), 13 in Table S1).^31^ Subsequently, the phosphorylated IRF3 induces a negative regulator *Socs1* expression (reactions 14 in Table S1). As we have reported, the activation of TLR7 can trigger MyD88‐dependent IRF‐mediated IFNα/β response to *plasmodium* infection in pDC, and this process might be potently inhibited by SOCS1 (reactions (15), (16), (17), (18), (19) in Table S1).^30^

The computational model was formulated using ordinary differential equations (ODEs) in MATLAB R2010a (MathWorks). The corresponding ODE model is described by the following equations:

(1) $$\frac{d[YM]}{\mathrm{dt}}=‐dYM\left[YM\right]$$

(2) $$\frac{d\;[acGAS]}{\mathrm{dt}}=kac\;\left[YM\right]\;\left(1‐\left[acGAS\right]\right)‐dac\;\;\left[acGAS\right]$$

(3) $$\frac{d\;[aSTING]}{\mathrm{dt}}=\frac{kaS\;((1+2kUSA)\;\;[acGAS])\;(1‐[aSTING])}{KaS+(1‐[aSTING])}‐daS\;\left[aSTING\right]$$

(4) $$\frac{d\;[aMDA5]}{\mathrm{dt}}=kaM5\;\left[YM\right]\;\left(1‐\left[aMDA5\right]\right)‐daM5\;\left[aMDA5\right]$$

(5) $$\frac{d\;[aMAVS]}{\mathrm{dt}}=\frac{kaMA\;((1+2kUSB)\;[aMDA5])\;(1‐[aMAVS])}{KaMA+(1‐[aMAVS])}‐daMA\;\left[aMAVS\right]$$

(6) $$\frac{d\;[aAIM2]}{\mathrm{dt}}=kaA\;\left[YM\right]\;\left(1‐\left[aAIM2\right]\right)‐daA\;\left[aAIM2\right]$$

(7) $$\frac{d\;[aNLRP3]}{\mathrm{dt}}=kaN\;\left[YM\right]\;\left(1‐\left[aNLRP3\right]\right)‐daN\;\left[aNLRP3\right]$$

(8) $$\begin{array}{c} \frac{d\;[caspase‐1]}{\mathrm{dt}}=\frac{kaAC\;[aAIM2]\;(1‐[caspase‐1])}{KaAC+(1‐[caspase‐1])}\\ +\frac{kaNC\;\;[aNLRP3]\;(1‐[caspase‐1])}{KaNC+(1‐[caspase‐1])}‐daC\;\left[caspase‐1\right] \end{array}$$

(9) $$\frac{d\;[aIL1R1]}{\mathrm{dt}}=kILR\;\left[caspase‐1\right]\;\left(\frac{kpIL1B\;[YM]}{KpIL1B+[YM]}\right)‐dILR\;\left[aIL1R1\right]$$

(10) $$\begin{array}{c} \frac{d[pIRF3]}{\mathrm{dt}}=\frac{kSI3[aSTING](1‐[pIRF3])}{KSI3+(1‐[pIRF3])}+\frac{kMI3[aMAVS](1‐[pIRF3])}{KMI3+(1‐[pIRF3])}\\ +\frac{kII3[aIL1R1](1‐[pIRF3])}{KII3+(1‐[pIRF3])}‐dpI3\left[pIRF3\right] \end{array}$$

(11) $$\frac{d\;[SOCS1]}{\mathrm{dt}}=\frac{kI3S\;{\left[pIRF3\right]}^{n1}}{KI3S+{\left[pIRF3\right]}^{n1}}‐dmS\;\left[SOCS1\right]$$

(12) $$\frac{d\;[aTLR7]}{\mathrm{dt}}=kaT7\;\;\left[YM\right]\;\left(1‐\left[aTLR7\right]\right)‐daT7\;\left[aTLR7\right]$$

(13) $$\frac{d\;[aMyD88]}{\mathrm{dt}}=\frac{kaM8\;[aTLR7]\;(1‐[aMyD88])}{KaM8+(1‐[aMyD88])}‐daM8\;\left[aMyD88\;\right]$$

(14) $$\frac{d\;[pIRF7]}{\mathrm{dt}}=\frac{kMI7\;(1‐[pIRF7])}{KMI7+(1‐[pIRF7])}{\left(\frac{\left[aMyD88\right]}{KSIM+[SOCS1]}\right)}^{n2}‐dI7\;\left[pIRF7\right]$$

(15) $$\frac{d\;[IFN\alpha ]}{\mathrm{dt}}=\frac{kIFN\alpha \;[pIRF7]}{KIFN\alpha +[pIRF7]}‐dIFN\alpha \;\left[IFN\alpha \right]$$

(16) $$\frac{d\;[IFN\beta ]}{\mathrm{dt}}=\frac{kIFN\beta \;[pIRF7]}{KIFN\beta +[pIRF7]}‐dIFN\beta \;\left[IFN\beta \right]$$

**FIGURE 1:**
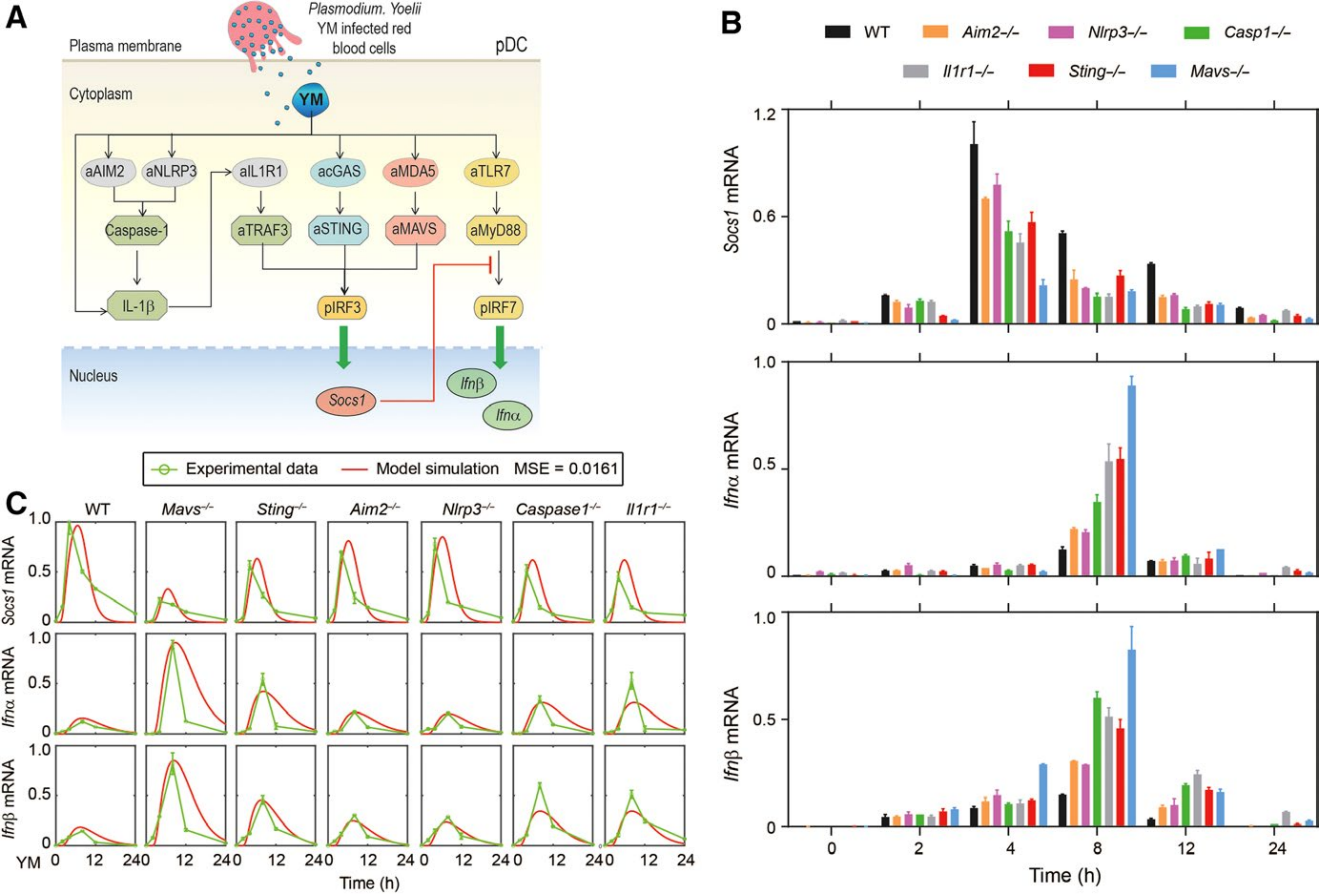
Modelling MAVS‐, STING‐ and Inflammasome‐mediated *Socs1* expression to inhibit MyD88‐dependent *Ifnα/β* response to YM infection. (A) Schematic representation of STING‐, MAVS‐ and inflammasome‐mediated *Socs1* expression to inhibit MyD88‐dependent type I IFN signalling in pDCs. (B) The WT, *Mavs^‐/‐^*, *Sting^‐/‐^*, *Aim2^‐/‐^*, *Nlrp3^‐/‐^*, *Caspase1^‐/‐^* and *Il1r1^‐/‐^* pDCs were stimulated with *plasmodium* gDNA + RNA + haemozoin for indicated times, and RNA from pDCs was isolated and used for expression *Socs1* and *Ifnα/β* by using qPCR. (C) Model simulations (red lines) fitted well with experimental data (green dots, showing in Figure 1B). Data are representatives of three independent experiments with similar results and plotted as mean ± SD

The unknown parameters were estimated using non‐linear least square method using genetic algorithm.^35^ The time course of *Socs1, Ifnα* and *Ifnβ* mRNA under 7 different conditions (WT, *Aim2^‐/‐^*, *Nlrp3^‐/‐^*, *Caspase1^‐/‐^*, *Il1r1^‐/‐^*, *Sting^‐/‐^* and *Mavs^‐/‐^*; Figure 1B) was integrated into the fitting using the following objective function:

(17) $$\hat{\theta }=\underset{\theta \hat{I}\Theta }{\arg\min}\sum_{i=1}^{m}\sum_{j=1}^{n_{i}}{\left(Y^{\text{sim}}\left({\text{cond}}_{i},t_{j};\theta \right)‐Y^{\exp}\left({\text{cond}}_{i},t_{j};\theta \right)\right)}^{2}$$

where *Y^sim^*(*cond_i_,t_j_,θ*) represents the simulated *Socs1, Ifnα* and *Ifnβ* mRNA at time‐point *t_j_* under the *i*‐th condition (*cond_i_*) with parameter set *θ*; *Y^exp^*(*cond_i_,t_j_,θ*) denotes the corresponding experimental data; and *Θ* indicates the parameter space.

Local sensitivity coefficient evaluates the systematic responses to an infinitesimal disturbance in nominal model parameters. The relative change of a derived systematic quantity M with respect to a relative change (1%) of parameter p is given as follows ^36^:

(18) $$S_{p_{i}}^{M}=\frac{p_{i}}{M}\cdot \frac{\partial M}{\partial p_{i}}$$

We perform the Bliss index to quantitatively identify whether the co‐administration of two parameters, such as kaMA with kILR, produces synergistic effects on *Socs1* expression. The index is defined by the following equation ^37^:

(19) $${\text{CI}}_{\text{Bliss}}\left(x1,x2\right)=\frac{O_{1}\left(x1\right)+O_{2}\left(x2\right)‐O_{1}\left(x1\right)\cdot O_{2}\left(x2\right)}{O_{12}\left(x1,x2\right)}$$

where O_1_(x1), O_2_(x2) and O_12_(x1,x2) are the relative *SOCS1* mRNA to kaMA (at a x1 fold of its initial value), kILR (at a x2 fold of its initial value) and their combination (at (x1,x2) multiplier), respectively. Therefore, CI_Bliss_ <1, CI_Bliss_ >1 and CI_Bliss_ = 1 denote synergistic, antagonistic and additive combination effects, respectively.

### 2.7 Statistical analysis

The results of all quantitative experiments are reported as mean ± SD of three independent experiments. Comparisons between groups for statistical significance were assessed with a two‐tailed Student's *t* test.

## 3 RESULTS

### 3.1 Mathematical model could quantitatively reproduce the dynamics of *Socs1* and *Ifnα/β* expression by YM treatment

According to previous studies and our experimental data, we concluded a schematic representation of the dynamic regulation of MAVS/STING, inflammasome and MyD88‐IRF7‐dependent type I IFN response to YM infection as shown in Figure 1A.^24^, ^30^, ^31^ During YM infection, the gDNA‐haemozoin complex triggers the AIM2 and NLRP3 inflammasome activation, which then initiates the IRF3‐dependent *Socs1* expression through IL‐1R1‐TRAF3‐IRF3‐mediated signalling. Besides, upon binding to *plasmodium* gDNA, the activation of cGAS and an unrevealed DNA sensor leads to recruit and active STING. Meanwhile, the activation of MDA5 and an unrevealed RNA sensor binding with *plasmodium* RNA evoke the activation and recruitment of adaptor protein MAVS. The STING‐ and MAVS‐mediated pathways subsequently induce the phosphorylation of IRF3 to further trigger the *Socs1* expression. At the same time, the *plasmodium* RNA also could activate the TLR7‐MyD88‐IRF7‐dependent type I IFN response, although this process was inhibited by SOCS1 through dynamic interaction between SOCS1 and MyD88.

A key question is how these pathways finely regulate the dynamic response of type I interferon to YM infection. To address this issue, we isolated pDCs from WT and gene deficient (*Mavs^‐/‐^*, *Sting^‐/‐^*, *Aim2^‐/‐^*, *Nlrp3^‐/‐^*, *Caspase1^‐/‐^* and *Il1r1^‐/‐^*) mice, treated the pDCs with *plasmodium* gDNA + RNA+haemozoin for indicated times (Figure 1B) and assessed the mRNA expression levels of *Socs1*, *Ifnα* and *Ifnβ*. We found that the peaked values of *Socs1* mRNA were dramatically decreased in *Mavs^‐/‐^* pDCs, modest decreased in *Sting^‐/‐^*, *Caspase1^‐/‐^* and *Il1r1^‐/‐^* pDCs, and slightly decreased in *Aim2^‐/‐^* and *Nlrp3^‐/‐^* pDCs, compared with WT pDCs (Figure 1B, top panel, Time = 4 hours). the expression levels of *Socs1* inversely correlated with levels of *Ifnα* and *Ifnβ* induction (Figure 1B). Strikingly, we found that at early stage (Time = 2 hours, top panel), the STING‐ and MAVS‐dependent signalling pathways play a predominant role in *Socs1* expression, whereas the inflammasome‐mediated pathway has greater influence on *Ifnβ* induction at late stage (Time = 12 hours, bottom panel) (Figure 1B). Based on these observations, we developed a computational model to illustrate the signalling strength‐dependent dynamic responses to YM infection. To estimate the unknown parameters of model, we adopted non‐linear least square method to minimize the sum of squared differences between experimental and simulated data by employing genetic algorithm.^35^ The mean squared error (MSE) is a measure that reflects the difference between experimental and simulated data, and the MSE is as small as possible. The MSE was 0.0161, which indicated that the temporal dynamics of simulated *Socs1* and *Ifnα/β* mRNA fitted well with experimental data under 7 different conditions (WT, *Mavs^‐/‐^*, *Sting^‐/‐^*, *Aim2^‐/‐^*, *Nlrp3^‐/‐^*, *Caspase1^‐/‐^* and *Il1r1^‐/‐^*; Figure 1C). Therefore, our mathematical modelling could reproduce temporal patterns of variables involved in dynamic regulatory networks of type I IFN response to YM infection.

### 3.2 Properties of MAVS‐, STING‐ and Inflammasome‐mediated signalling contribute diverse characteristics in *Socs1* and *Ifnα/β* expression

**FIGURE 2:**
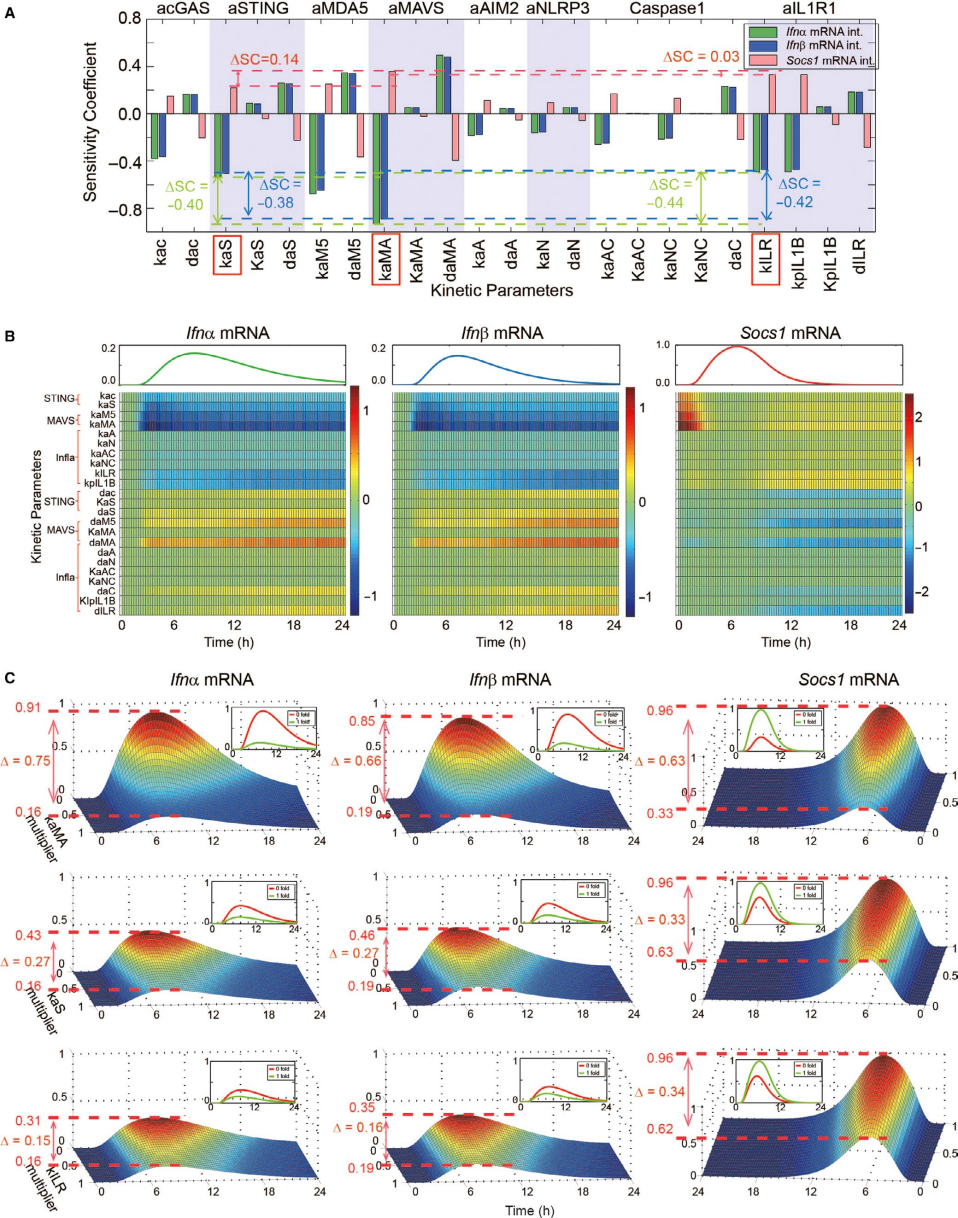
Properties of MAVS‐, STING‐ and Inflammasome‐mediated signalling contribute diverse characteristics in anti‐malaria response. (A‐B) Local sensitivity analysis of integrated value (A) and time course (B) of *Socs1* and *Ifnα/β* mRNA with respect to each kinetic parameter. (C) Temporal trajectories of *Socs1* and *Ifnα/β* mRNA with varying value of kaS, kaMA or kILR. Note that the kaS, kaMA and kILR were rescaled to indicate relative changes to the simulated values

Our experimental results in Figure 1B suggested that the STING‐, MAVS‐ and inflammasome‐mediated signalling pathways might have distinct influence on *Socs1* and *Ifnα/β* mRNA dynamics. To quantitatively identify the precise role of MAVS‐, STING‐ and inflammasome‐mediated signalling in TLR7‐MyD88‐IRF7‐dependent type I IFN response to YM infection, we performed the local sensitivity analysis for all kinetic parameters to calculate sensitivities of integrated values of *Socs1* and *Ifnα/β* mRNA (Figure 2A). As the local sensitivity analysis could quantitatively denote the contribution of each parameter, we found that parameters involved in MAVS activation had the greatest impact on *Socs1* and *Ifnα/β* expression, whereas parameters involved in STING and IL1R1 activation had similar impact on *Socs1* and *Ifnα/β* expression. Besides, the sensitivity analysis of temporal pattern of *Socs1* and *Ifnα/β* mRNA also showed that during *Plasmodium* infection, the parameters involved in MAVS activation were the most sensitive, whereas were more sensitive at early stage than late (eg kaMA); parameters involved in STING activation were sensitive at the early stage, but rapidly weakened (eg kaS); and parameters involved in inflammasome activation were less sensitive at the early stage than late, whereas there is sustained modest sensitivity at late stage (eg kILR) (Figure 2B). Furthermore, the simulated trajectories of *Socs1* and *Ifnα/β* mRNA also indicated that the decrease of kaMA significantly reduced the *Socs1* expression but enhanced *Ifnα/β* production, whereas the change of kaS and kILR had modest and similar influence on *Socs1* and *Ifnα/β* expression (Figure 2C). Interestingly, as the value of kaS or kILR shifted from 1 to 0, the peak value of *Socs1* mRNA decreased by −0.33 or −0.34, respectively, which was almost equal. However, the peak value of *Ifnα/β* mRNA increased by 0.27/0.27 or 0.15/0.16, respectively, which was obviously different (Figure 2C). Taken together, these results demonstrated that during the YM infection, MAVS‐mediated signalling plays a predominant role in inducing *Socs1* expression and inhibiting type I IFN response, whereas STING‐ and inflammasome‐mediated pathways have similar but less contribution than MAVS‐dependent signalling to *Socs1* expression. In addition, STING‐mediated pathway has slightly more influence on *Ifnα/β* expression than inflammasome.

### 3.3 The YM dose could significantly affect the difference of resistance to YM infection among MAVS, STING and inflammasome deficiency

**FIGURE 3:**
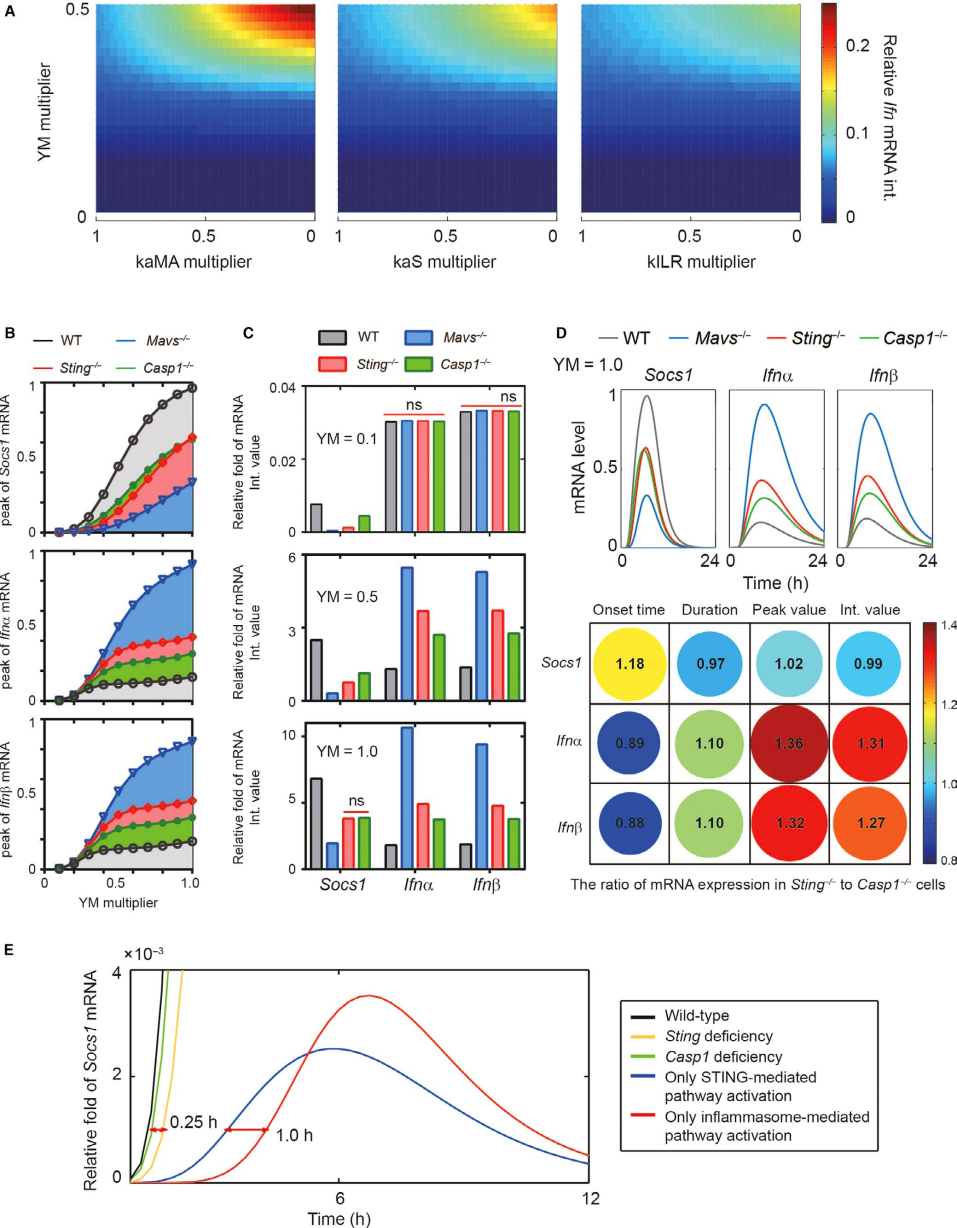
MAVS, STING and Inflammasome deficiency have distinct resistance to YM infection. (A) The integrated value of *Socs1* and *Ifnα/β* mRNA with varying dose of YM and value of kaMA, kaS or kILR. Note that the YM, kaS, kaMA and kILR were rescaled to indicate relative changes to the simulated values. (B‐C) The peaked (B) and integrated (C) value of *Socs1* and *Ifnα/β* mRNA with increasing dose of YM under WT, *Caspase1* ^‐/‐^, *Sting* ^‐/‐^ or *Mavs* ^‐/‐^ conditions. (D) The time course of *Socs1* and *Ifnα/β* mRNA under WT, *Caspase1* ^‐/‐^, *Sting* ^‐/‐^ or *Mavs* ^‐/‐^ conditions. (E) The temporal dynamics of *Socs1* mRNA under WT, *Caspase1* ^‐/‐^, *Sting* ^‐/‐^, only STING‐mediated pathway activation or only inflammasome‐mediated pathway activation

Our previous studies have shown that the MAVS deficiency mice confer stronger inhibition of *Socs1* expression, leading to higher level of *Ifnα/β* production, and consequently stronger resistance to YM challenging than Caspase1 deficiency.^31^ To further qualitatively and quantitatively determine the resistance of MAVS, STING or Caspase1 deficiency to YM infection, we in silico‐varied the YM doses and kaMA/kaS/kILR values simultaneously. Analysis of *Ifn*s mRNA int. phase spaces showed that at high dosage of YM treatment, the variation in kaMA value had greatest contribution to inhibit *Ifn*s expression; and the impact of variation in kaS value on *Ifn*s production was more significant than kILR, whereas upon low dosage YM treatment, the change of kaMA, kaS or kILR value did not substantially influence the type I IFN response to YM infection (Figure 3A). Thus, we hypothesized that the YM dose might affect the difference of contribution among MAVS‐, STING‐ and inflammasome‐mediated pathways on MyD88‐IRF7‐dependent type I IFN induction. Analysing the dose dependence of *Socs1* and *Ifnα/β* mRNA peaked and integrated values in WT, *Mavs* ^‐/‐^, *Sting* ^‐/‐^ or *Caspase1* ^‐/‐^ pDCs, we found that in low‐dose YM infection (eg YM = 0.1), there were similar production of MyD88‐IRF7‐dependent type I IFN response, while diverse *Socs1* expression among all types pDCs (Figure 3B‐C). However, treating with high dosage of YM infection (eg YM = 1.0), the levels of *Ifnα/β* mRNA were different among various knockout pDCs, whereas interestingly, the levels of *Socs1* mRNA were almost equal in *Caspase1* ^‐/‐^ and *Sting* ^‐/‐^ pDCs (Figure 3B‐C), which was consistent with the description in Figure 2C. Collectively, our model simulation predicts that at the low doses of YM infection, the STING, MAVS and inflammasome deficiencies have similar resistance to YM challenging, whereas at the high doses of YM infection, MAVS signalling contributes more to the resistance to YM infection than STING or Caspase‐1.

To further reveal why upon high dosage of YM infection (eg YM = 1.0), the levels of *Socs1* mRNA were similar, but the *Ifnα/β* expression was substantially different in *Caspase1* ^‐/‐^ and *Sting* ^‐/‐^ pDCs, and we quantitatively analysed the time course of *Socs1* and *Ifnα/β* mRNA (Figure 3D). The simulated results indicated that the duration, peak values, and integrated values of *Socs1* mRNA were barely different in *Caspase1* ^‐/‐^ and *Sting* ^‐/‐^ pDCs, but the onset time of *Socs1* expression in *Caspase1* ^‐/‐^ pDCs was earlier than in *Sting* ^‐/‐^ pDCs, which resulted in a latter onset time of *Ifnα/β* mRNA in *Caspase1* ^‐/‐^ than in *Sting* ^‐/‐^ pDCs. Thus, we supposed that the STING‐mediated *Socs1* expression might earlier than Caspase‐1. Then, we compared the onset time of *Socs1* expression between Sting and Casp1 deficiencies mice, or between only STING‐ and only CASP1‐mediated pathways activation (Figure 3E). The in silico results verified that the onset time of *Socs1* mRNA induced by STING‐mediated pathway was earlier 1.0 hour than CASP1‐mediated signalling. In summary, these results suggested that the *Socs1* expression induced by STING‐ mediated was earlier than inflammasome, leading to latter onset time of IRF7‐ MyD88‐dependent type I IFN response to YM infection.

### 3.4 The synergistic effect of MAVS‐, STING‐ and inflammasome‐mediated pathways on *Socs1* expression is distinct for diverse time and stimulus

**FIGURE 4:**
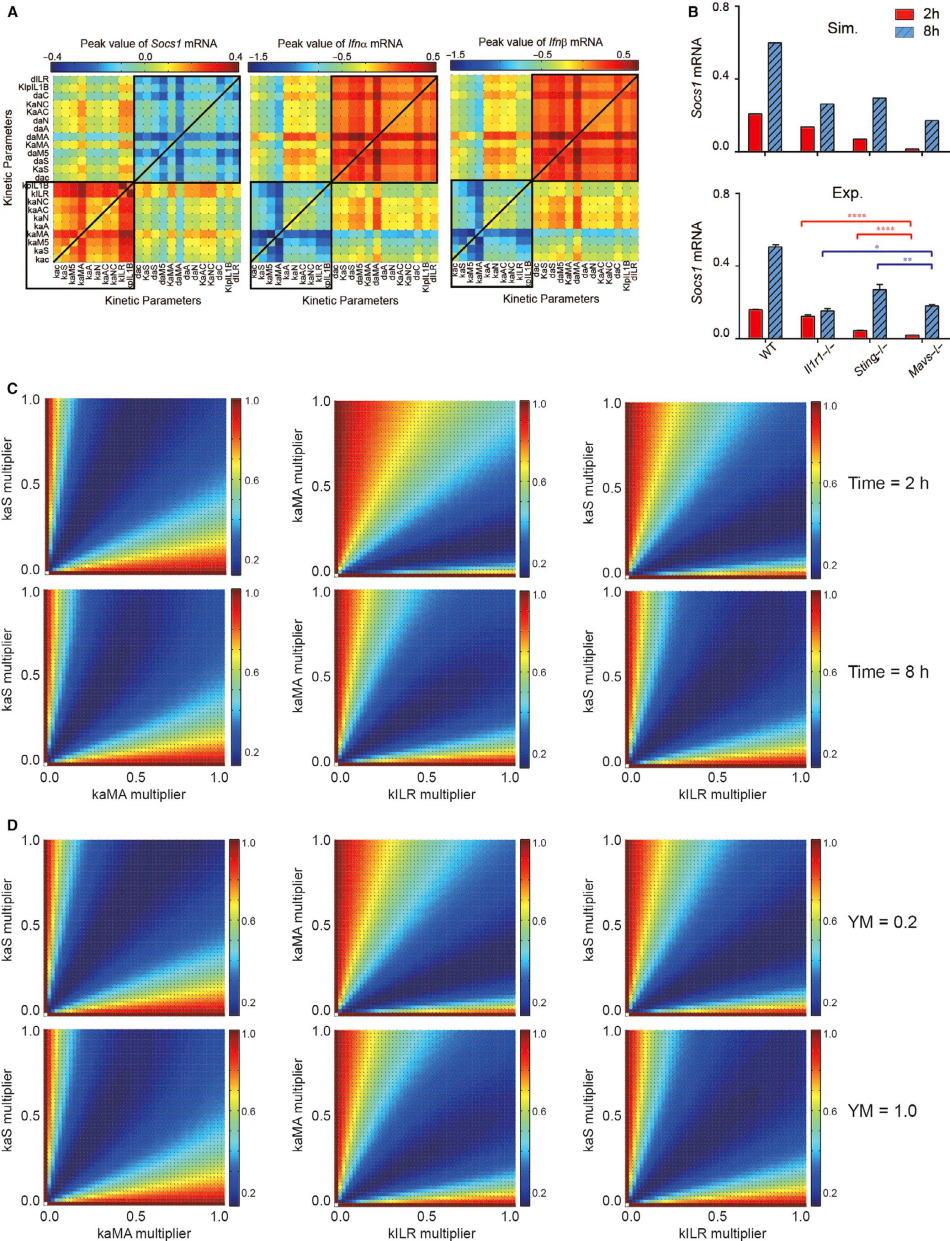
The combination of MAVS‐, STING‐ and Inflammasome‐mediated pathways has synergistic effect on *Socs1* expression. (A) Two‐parameter sensitivity analysis of integrated values of *Socs1* and *Ifns* mRNA with respect to the combinatorial variations in parameter values. (B) Induction of *Socs1* mRNA by YM treatment. The red and blue bars represent *Socs1* expression after 2 and 8 h of stimulation, respectively. Data were presented as the mean ± SD of three independent experiments. (C‐D) Synergy prediction about *Socs1* expression at 2 and 8 h (C), or integrated value of *Socs1* mRNA infected by 0.2‐ and 1.0‐fold of YM dose (D), on dual combinations of kaS and kaMA, kaMA and kILR, as well as kaS and kILR, based on Bliss combination index

To thoroughly uncover how the MAVS‐, STING‐ and inflammasome‐mediated pathways combine with each other to impact the *Socs1* and *Ifnα/β* expression, we conducted a two‐parameter sensitivity analysis (Figure 4A). This result revealed that the combination of parameters involved in STING, MAVS and inflammasome activation might cooperatively promote the *Socs1* expression and suppress the IRF7‐ MyD88‐ dependent type I IFN response to YM infection (black square). As we have mentioned above that MAVS‐mediated signalling has a predominant contribution, whereas STING‐ and inflammasome‐mediated pathways have similar but less contribution to *Socs1* and *Ifnα/β* expression, we then implemented the local sensitivity analysis of Socs1 and *Ifnα/β* expression at different time to mechanistically investigate the dynamic cooperation of these pathways (Figure S1A). We found that after YM treatment, at 2 and 4 hour, the difference of coefficient between kaMA (rate constant of MAVS activation) and kaS (rate constant of STING activation) or kILR (rate constant of IL1R1 activation) was 0.80 or 1.31 and −0.53 or −0.76, respectively, whereas at 8 and 16 hour, the difference was 0.12 or −0.04 and −0.36 or −0.32, respectively. Besides, both model prediction and experimental validation suggested that the difference of *Socs1* expression between in *Mavs^‐/‐^* and *Sting^‐/‐^* or *Il1r1^‐/‐^* pDCs were greater at early stage (time = 2 hours) than at late stage (time = 8 hours) (Figure 4B). These results showed that MAVS‐mediated signalling plays a predominant role in *Socs1* and *Ifns* expression at early phase, and then cooperates with STING‐ and inflammasome‐mediated pathways. Furthermore, the synergistic effect analysis, employed Bliss index,^37^ also shows that the synergistic/antagonistic effects of MAVS‐, STING‐ and Inflammasome‐mediated pathways on *Socs1*/*Ifns* expression were weaker at early phase (time = 2 or 4 hours for *Socs1* or *Ifns* expression, respectively) than late phase (time = 8 or 16 hours for *Socs1* or *Ifns* expression, respectively) (Figure 4C and Figure S1B). In addition, our in silico results exhibited that the synergistic/antagonistic effects of MAVS‐, STING‐ and Inflammasome‐mediated signalling on *Socs1*/*Ifns* expression were better in pDCs treated with high dosage YM than low dose (Figure 4D and Figure S1C). Therefore, our results suggested that the synergistic/antagonistic effects of MAVS‐, STING‐ and Inflammasome‐mediated signalling on *Socs1*/*Ifns* expression are distinct for diverse stimulation time or dosage.

## 4 DISCUSSION

As aberrant type I interferon response may result in many autoimmune diseases, tight modulation of type I IFN signalling is critical for maintaining homoeostasis of immune system after microbial invasion.^38^, ^39^ Accumulating evidence indicates that the type I IFN signalling plays an important role in anti‐malaria.^40^, ^41^, ^42^ Therefore, understanding the dynamic regulation on type I IFN may shed light on effective therapeutics for malaria infection. This study employed systems biology approach to systematically and comprehensively investigate the exact role of multiple pathways in the dynamic control of type I IFN response to YM infection.

We have reported that MAVS‐, STING‐ and inflammasome‐mediated pathways cooperatively induce *socs1* expression to inhibit TLR7‐MyD88‐IRF7‐dependent type I interferon response to YM challenging.^30^, ^31^ However, it is still lack of systematic and quantitative analysis of underlying mechanisms in precise regulatory mechanisms of type I interferon response to YM infection. Through the mathematical modelling, we demonstrated that (a) during the YM infection, MAVS‐mediated signalling plays a predominant role in *Socs1* expression, whereas STING‐ and inflammasome‐mediated pathways have similar but less contribution than MAVS‐dependent signalling to induce *Socs1* expression, (b) the YM dose could significantly affect the difference of resistance to YM infection among MAVS, STING and inflammasome deficiency, and (c) the STING‐mediated pathway works earlier than inflammasome to enhance SOCS1 production. Furthermore, (d) the synergistic or antagonistic effect of these three pathways on *Socs1* or *Ifnα/β* expression is distinct for varying time and stimulus dose.

Our previous study has shown that the *Socs1* or *Ifnα/β* expression significantly decreased or increased in *Mavs^‐/‐^* pDCs, respectively, whereas modest varied in *Casp1^‐/‐^* pDCs. We further identified that MAVS deficiency confers stronger resistance to YM infection than Casp1 deficiency.^31^ Our model further qualitatively and quantitatively revealed that upon low dosage of YM infection, the *Ifnα/β* expression was similar among *Mavs^‐/‐^*, *Sting^‐/‐^* and *Casp1^‐/‐^* pDCs, which suggested that all knockout mice had similar resistance. However, at high dosage of YM stimulus, MAVS deficiency conferred the strongest resistance, STING deficiency conferred modest resistance, and Casp1 deficiency have slight resistance. Notably, in high‐dose infection (YM = 1), STING‐ and Caspase1‐mediated signalling contributed equally to induce *Socs1* expression, but diverse type I IFN production, which may arise from earlier onset time of *Socs1* mRNA triggered by STING‐mediated pathway than Casp1‐dependent signalling.

Collectively, by incorporating mathematical modelling and experimental results, our study not only revealed the specific contribution of MAVS‐, STING‐, and inflammasome‐mediated signalling for inducing *Socs1* expression to inhibit MyD88 dependent *Ifnα/β* expression in pDC, but also demonstrated the distinct synergistic effect of these pathways on *Socs1* expression with varying YM dose or stimulated time. Besides, our model analysis indicated that the YM dose could significantly affect the difference of resistance to YM infection among MAVS‐, STING‐, and inflammasome‐deficient mice. Furthermore, we attributed the distinct impacts of STING‐ and Casp1‐mediated signalling on type I IFN response to diverse onset time of *Socs1* expression induced by above signalling. Thus, our findings may provide mechanistic insights into dynamic regulation of IRF7‐dependent type I IFN response to YM infection, and provide impetus to orchestrate effective innate immune response to YM challenging by manipulate multiple pathways.

## CONFLICTS OF INTEREST

The authors declare no conflict(s) of interest.

## AUTHOR CONTRIBUTIONS

**Chunmei Cai:** Conceptualization (equal); Data curation (equal); Formal analysis (lead); Funding acquisition (equal); Investigation (equal); Methodology (lead); Project administration (equal); Resources (equal); Software (equal); Supervision (equal); Validation (equal); Visualization (lead); Writing‐original draft (equal); Writing‐review & editing (equal). **Xiao Yu:** Conceptualization (equal); Data curation (equal); Formal analysis (supporting); Funding acquisition (equal); Investigation (equal); Methodology (supporting); Project administration (equal); Resources (equal); Software (equal); Supervision (equal); Validation (equal); Visualization (supporting); Writing‐original draft (equal); Writing‐review & editing (equal).

## Supporting information

App S1

## Data Availability

The data sets used and/or analysed during the current study are available from the corresponding author upon reasonable request.
