# Supplementary material for: A mathematic model to reveal delicate cross‐regulation between MAVS/STING, inflammasome and MyD88‐dependent type I interferon signalling
Source: J Cell Mol Med. 2020 Sep 3;24(19):11535–45. doi: 10.1111/jcmm.15768 (PMC7576308; doi:10.1111/jcmm.15768)
Supplement: Supplementary file 1 — App S1 [file JCMM-24-11535-s001.pdf]

Online Supplementary Files

A mathematic model to reveal delicate cross-regulation between MAVS/STING, inflammasome and MyD88 dependent type I interferon signaling

Chunmei Cai,[1,2*] and Xiao Yu[3,4*]

[1]Research Center for High Altitude Medicine, School of Medical, Qinghai University, [2]Key Laboratory of Application and Foundation for High Altitude Medicine Research in Qinghai Province, Xining, P. R. China.

[3]Department of Immunology, School of Basic Medical Sciences, [4]Guangdong Provincial Key Lab of Single Cell Technology and Application, Southern Medical University, Guangzhou 510515, Guangdong, P. R. China.

* Corresponding author

E-mail: caicm@qhu.edu.cn （CC）

xiaoyu523@smu.edu.cn (XY)

Content

I Supplementary Figures······························2

Figure S1 Related to Figure 4. The combination of MAVS-, STING-, and Inflammasome-mediated pathways has antagonistic effect on *Ifns* expression.························2

II Supplementary Tables······························3

Table S1 Related to Figure 1: Reactions and values of variables and parameters involved in the mathematical model.······························3

I. Supplementary Figures

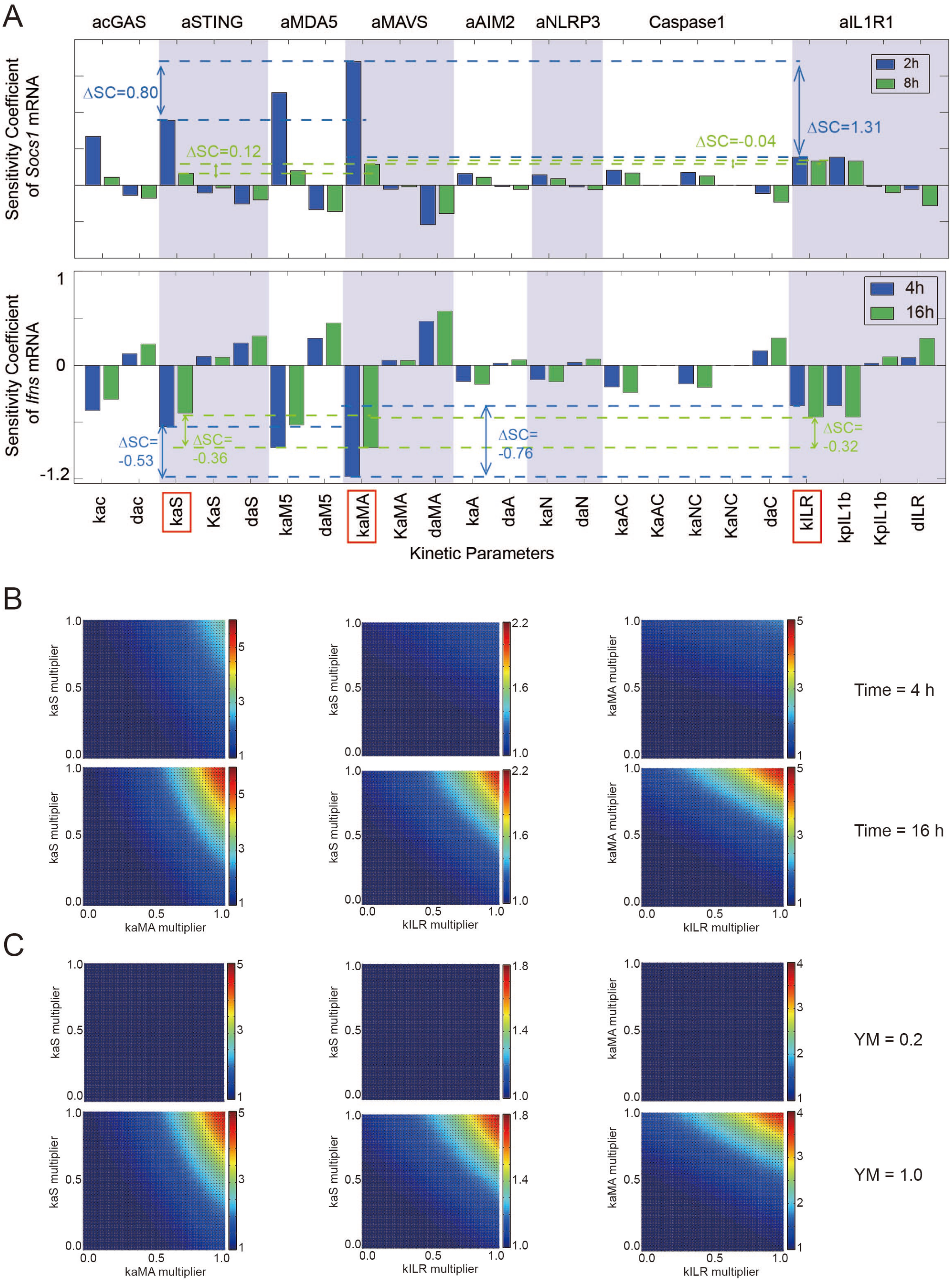

Supplementary Figure S1. The combination of MAVS-, STING-, and Inflammasome-mediated pathways has antagonistic effect on *Ifns* expression. (A) Local sensitivity analysis of *Socs1* and *Ifns* mRNA with respect to each kinetic parameter. The blue and green bars represent 2h/4h and 8h/16h. (B-C) Synergy prediction about *Ifns* expression at 4 and 16 hour (B), or integrated value of *Ifns* mRNA infected by 0.2 and 1.0 fold of YM dose (C), on dual combinations of kaS and kaMA, kaMA and kILR, as well as kaS and kILR, based on Bliss combination index.

II. Supplementary Tables

Supplementary Table S1: Reactions and values of variables and parameters involved in the mathematical model.

	Reactions	Symbols	Values	Description
1	$YM \xrightarrow{dYM} null$	YM	1	The normalized initial value of YM
		dYM	0.8861	Rate constant of YM degradation
2	$YM + cGAS \underset{dac}{\overset{kac}{\rightleftarrows}} YM + acGAS$	kac	0.9722	Rate constant of cGAS activation induced by YM
		dac	0.3181	Rate constant of acGAS deactivation
3	$acGAS + STING \underset{daS}{\overset{kaS,kUSA,KaS}{\rightleftarrows}} acGAS + aSTING$	kaS	0.9502	Rate constant of STING activation induced by acGAS and unrevealed sensor A (aUSA)
		kUSA	0.5431	Rate constant of USA activation dynamics along with acGAS
		KaS	0.0745	Half-saturation constant of STING activation induced by by acGAS and aUSA
		daS	0.5215	Rate constant of aSTING deactivation
4	$YM + MDA5 \underset{daM5}{\overset{kaM5}{\rightleftarrows}} YM + aMDA5$	kaM5	0.9991	Rate constant of MDA5 activation induced by YM
		daM5	0. 4171	Rate constant of aMDA5 deactivation
5	$aMDA5 + MAVS \underset{daMA}{\overset{kaMA,kUSB,KaMA}{\rightleftarrows}} aMDA5 + aMAVS$	kaMA	0. 9961	Rate constant of MAVS activation induced by aMDA5 and unrevealed sensor B (aUSB)
		kUSB	0. 6001	Rate constant of USB activation dynamics along with aMDA5
		KaMA	0. 0147	Half-saturation constant of MAVS activation induced by by aMDA5 and aUSB
		daMA	0. 6001	Rate constant of aMAVS deactivation
6	$YM + AIM2 \underset{daA}{\overset{kaA}{\rightleftarrows}} YM + aAIM2$	kaA	0. 9912	Rate constant of AIM2 activation induced by YM
		daA	0. 2037	Rate constant of aAIM2 deactivation
7	$YM + NLRP3 \underset{daN}{\overset{kaN}{\rightleftarrows}} YM + aNLRP3$	kaN	0. 8722	Rate constant of NLRP3 activation induced by YM
		daN	0. 3181	Rate constant of aNLRP3 deactivation
8	$pro-Caspase-1 + aAIM2 \underset{daC}{\overset{kaAC,KaAC}{\rightleftarrows}} aAIM2 + Caspase-1$	kaAC	0.9981	Rate constant of pro-Caspase-1 maturation induced by aAIM2
		KaAC	0.0012	Half-saturation constant of pro-Caspase-1 maturation induced by

				aAIM2
		daC	0.7543	Rate constant of Caspase-1 degradation
9	$\text{pro}-\text{Caspase}-1 + \text{aNLRP3} \underset{\text{daC}}{\overset{\text{kaNC,KaNC}}{\rightleftarrows}} \text{aNLRP3} + \text{Caspase}-1$	kaNC	0.9920	Rate constant of pro-Caspase-1 maturation induced by aNLRP3
		KaNC	0.0011	Half-saturation constant of pro-Caspase-1 maturation induced by aNLRP3
10	$\text{YM} + \text{Caspase}-1 + \text{IL1R1} \underset{\text{dILR}}{\overset{\text{kILR,kpIL1B,KpIL1B}}{\rightleftarrows}} \text{YM} + \text{Caspase}-1 + \text{aIL1R1}$	kILR	0.9387	Rate constant of IL1R1 signaling activation induced by Caspase1
		kpIL1B	0.6911	Rate constant of IL-1β expression induced by YM
		KpIL1B	0.0139	Half-saturation constant of IL-1β expression induced by YM
		dILR	0.3192	Rate constant of IL1R1 signaling deactivation
11	$\text{aSTING} + \text{IRF3} \underset{\text{dpI3}}{\overset{\text{kSI3,KSI3}}{\rightleftarrows}} \text{aSTING} + \text{pIRF3}$	kSI3	0.3394	Rate constant of IRF3 phosphorylation induced by aSTING
		KSI3	0.0131	Half-saturation constant of IRF3 phosphorylation induced by aSTING
		dpI3	0.9886	Rate constant of pIRF3 dephosphorylation
12	$\text{aMAVS} + \text{IRF3} \underset{\text{dpI3}}{\overset{\text{kMI3,KMI3}}{\rightleftarrows}} \text{aMAVS} + \text{pIRF3}$	kMI3	0.5280	Rate constant of IRF3 phosphorylation induced by aMAVS
		KMI3	0.0051	Half-saturation constant of IRF3 phosphorylation induced by aMAVS
13	$\text{aIL1R1} + \text{IRF3} \underset{\text{dpI3}}{\overset{\text{kII3,KII3}}{\rightleftarrows}} \text{aIL1R1} + \text{pIRF3}$	kII3	0.3419	Rate constant of IRF3 phosphorylation induced by aIL1R1
		KII3	0.0105	Half-saturation constant of IRF3 phosphorylation induced by aIL1R1
14	$\text{pIRF3} \underset{\text{dmS}}{\overset{\text{kI3S,KI3S,n1}}{\rightleftarrows}} \mathit{SOCS}1 + \text{pIRF3}$	kI3S	0.7737	Rate constant of *Socs1* expression induced by pIRF3
		KI3S	0.2379	Half-saturation constant of *Socs1* expression induced by pIRF3
		n1	4	Hill coefficient of *Socs1* expression induced by pIRF3
		dmS	0.5167	Rate constant of *Socs1* mRNA degradation
15	$\text{YM+TLR7} \underset{\text{daT7}}{\overset{\text{kaT7}}{\rightleftarrows}} \text{YM+aTLR7}$	kaT7	0.9730	Rate constant of TLR7 activation induced by YM
		daT7	0.3020	Rate constant of aTLR7 deactivation
		kaM8	0.9720	Rate constant of MyD88 activation induced by aTLR7

16	aTLR7+MyD88 $\xrightleftharpoons[\text{daM8}]{\text{kaM8,KaM8}}$ aTLR7+aMyD88	KaM8	0.1299	Half-saturation constant of MyD88 activation induced by aTLR7
		daM8	0.5843	Rate constant of aMyD88 deactivation
17	aMyD88+IRF7 $\xrightleftharpoons[\text{dI7}]{\text{kMI7,KMI7,KSIM,n2}}$ aMyD88+pIRF7	kMI7	0.9843	Rate constant of IRF7 activation induced by aMyD88
		KSIM	0.4398	Half-saturation constant of inhibition on aMyD88 dynamics by SOCS1
		KMI7	0.5651	Half-saturation constant of IRF7 activation induced by aMyD88
		n2	4	Hill coefficient of inhibition on aMyD88 dynamics by SOCS1
		dI7	0.3261	Rate constant of pIRF7 dephosphorylation
18	pIRF7 $\xrightleftharpoons[\text{dIFN}\alpha]{\text{kIFN}\alpha\text{,KIFN}\alpha}$ pIRF7+$IFN\alpha$	kIFNα	0.7753	Rate constant of *Ifnα* expression induced by pIRF7
		KIFNα	0.3961	Half-saturation constant of *Ifnα* expression induced by pIRF7
		dIFNα	0.2333	Rate constant of *Ifnα* mRNA degradation
19	pIRF7 $\xrightleftharpoons[\text{dIFN}\beta]{\text{kIFN}\beta\text{,KIFN}\beta}$ pIRF7+$IFN\beta$	kIFNβ	0.6526	Rate constant of *Ifnβ* expression induced by pIRF7
		KIFNβ	0.2196	Half-saturation constant of *Ifnβ* expression induced by pIRF7
		dIFNβ	0.3331	Rate constant of *Ifnβ* mRNA degradation
